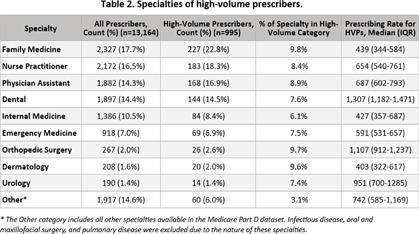# Leveraging Medicare Part D Data for Antibiotic Stewardship: Peer Comparison Feedback to High-Volume Prescribers in Minnesota

**DOI:** 10.1017/ash.2025.245

**Published:** 2025-09-24

**Authors:** Madeline Powers, Susan Gerbensky Klammer, Ruth Lynfield

**Affiliations:** 1 Minnesota Department of Health; 2 Minnesota Department of Health; 3 Minnesota Dept of Health

## Abstract

**Background:** Older adults are prescribed more antibiotics than younger populations and face increased risks of antibiotic-related adverse events. Identifying high-volume prescribers (HVPs) through Medicare Part D (MPD) datasets and engaging them through targeted intervention, such as peer-comparison audit and feedback, is a way to impact antibiotic prescribing. **Methods:** We analyzed the 2022 publicly available Centers for Medicare & Medicaid Services MPD Prescribers by Provider dataset to summarize the data overall and identify HVPs within each specialty. HVPs were classified as prescribers in the top 10% of antibiotic prescribing by volume within their specialty. Prescribers with 1,316 Minnesota prescribers in 2022 were considered HVPs (top 10% by volume by specialty). After removing certain specialties and those with low prescribing rates, 995 HVPs met criteria and were mailed feedback letters, with 4.32% (43 letters) lost to follow-up. These HVPs were responsible for 28.7% of antibiotic prescriptions for Minnesota’s MPD beneficiaries in 2022. The median antibiotic prescribing rate of these HVPs was 1.8 times higher than that of lower-volume prescribers (Table 1) (p To date, 18 letter recipients responded to the feedback survey, with 22.2% intending to review their current prescribing habits, 22.2% reflecting that there is room for improvement in their prescribing, and 55.6% have accessed or intend to access AS resources. **Conclusion:** This audit and feedback initiative demonstrated that the MPD dataset can be used as a low-cost method to provide peer-comparison feedback to HVPs. By reaching providers responsible for nearly 30% of antibiotic prescriptions among MPD beneficiaries in Minnesota, this intervention has potential to influence prescribing behaviors. Further work will evaluate feedback and focus on specific provider specialties and drug classes.

**Figure:**
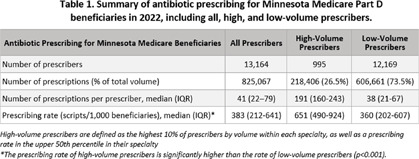

**Figure:**